# QSLiMFinder: improved short linear motif prediction using specific query protein data

**DOI:** 10.1093/bioinformatics/btv155

**Published:** 2015-03-19

**Authors:** Nicolas Palopoli, Kieren T. Lythgow, Richard J. Edwards

**Affiliations:** ^1^Centre for Biological Sciences, University of Southampton, Southampton, UK,; ^2^Public Health England, London, UK,; ^3^Institute for Life Sciences, University of Southampton, Southampton, UK and; ^4^School of Biotechnology and Biomolecular Sciences, University of New South Wales, Sydney, NSW, Australia

## Abstract

**Motivation:** The sensitivity of *de novo* short linear motif (SLiM) prediction is limited by the number of patterns (the motif space) being assessed for enrichment. QSLiMFinder uses specific query protein information to restrict the motif space and thereby increase the sensitivity and specificity of predictions.

**Results:** QSLiMFinder was extensively benchmarked using known SLiM-containing proteins and simulated protein interaction datasets of real human proteins. Exploiting prior knowledge of a query protein likely to be involved in a SLiM-mediated interaction increased the proportion of true positives correctly returned and reduced the proportion of datasets returning a false positive prediction. The biggest improvement was seen if a short region of the query protein flanking the interaction site was known.

**Availability and implementation:** All the tools and data used in this study, including QSLiMFinder and the SLiMBench benchmarking software, are freely available under a GNU license as part of SLiMSuite, at: http://bioware.soton.ac.uk.

**Contact:** richard.edwards@unsw.edu.au

**Supplementary information:** Supplementary data are available at *Bioinformatics* online.

## 1 Introduction

All biological processes are underpinned by protein–protein interactions (PPI). To understand the ‘interactome’, we must know how PPI are regulated in time and space to produce biological functions (Tuncbag *et al*., 2009). An emerging field of biology is the study of the role in PPI networks of intrinsically disordered protein regions (Babu *et al.*, 2011; Tompa, 2011), which lack a stable (unbound) three-dimensional structure. Of particular interest, short linear motifs (SLiMs) mediate an important subset of the cell’s disordered PPI via domain-motif interactions (Neduva and Russell, 2005; Pancsa and Fuxreiter, 2012; Russell and Gibson, 2008). SLiMs are typically 2–15 amino acids in length with fewer than six (and as few as two) functionally specific residues (Davey *et al*., 2012a). SLiMs are involved in an incredibly diverse range of biological processes, including cell cycle, cell signalling, post-translational modification, subcellular localization, gene expression, membrane binding, protein folding, cell adhesion and cell death, with over 200 annotated classes (Dinkel *et al*., 2014). SLiMs usually bind with low affinity, making them ideal for quick or transient responses, and are likely to be particularly enriched in signalling pathways (Diella *et al*., 2008).

The small protein sequence signature of SLiMs, combined with their low affinity PPI, makes experimental discovery difficult. Considerable attention has therefore been given to computational methods for SLiM prediction (Davey *et al*., 2010a; Edwards and Palopoli, 2015). These same features confer evolutionary plasticity on SLiM-mediated PPI and enable high functional density, which is frequently exploited by pathogens to hijack host cellular processes (Davey *et al*., 2011). Convergent (i.e*.* independent) evolution is also prevalent within species. Consequently, identifying over-represented motifs by explicitly modelling convergent evolution is among the most successful approaches for *de novo* prediction of SLiMs from protein sequences and PPI data (Davey *et al*., 2006, 2010a , 2010c; Edwards *et al*., 2007, 2012; Neduva and Russell, 2005, 2006). Of these, SLiMFinder was the first to introduce a robust (if slightly conservative) statistical model for *de novo* SLiM prediction that accounted for both the evolutionary relationships within the data (i.e*.* shared motifs due to homology) and the size of the motif space being search (i.e*.* the number of patterns being assessed for enrichment) (Davey *et al*., 2010b; Edwards *et al*., 2007).

The SLiMChance statistical model gives very high specificity predictions on benchmarking data (Edwards *et al*., 2007), making it suitable for large-scale analyses (Edwards *et al*., 2012). However, the specificity of SLiMChance is achieved at the expense of prediction sensitivity because the number of patterns being assessed—the motif space—is typically very large. Even without undefined positions, there are 20*^L^* possible patterns for a SLiM of length *L*, which demands a large multiple testing correction on enrichment statistics.

A second limitation of searching for over-representation in PPI datasets derives from the nature of the interactome itself. The search strategy makes the implicit assumption that any observed over-representation is causally linked to the reason for assembling that dataset, e.g*.* analysing proteins with a common interaction partner, assumes over-representation due to an interaction between that partner and the enriched motif. In reality, motifs can be enriched due to overlapping sets of shared PPI and/or proteome-wide motif enrichment (Edwards *et al*., 2012). Analysing a whole interactome by correlating motif presence/absence with PPI partners might offset this issue to some extent. FIRE-pro, for example, uses mutual information and network randomizations to identify SLiMs associated with PPI partners or biological processes/functions (Lieber *et al*., 2010). However, these approaches need to analyse full interactomes, making them computationally challenging and unable to fully correct for protein homology. Similarly, interactome-wide analyses and using random assemblies of proteins can identify recurring motifs (Edwards *et al*., 2012) but are not applicable to individual datasets of proteins.

Here, we present QSLiMFinder (‘Query’ SLiMFinder), which has been developed as an extension of SLiMFinder to explicitly harness additional information from interaction data in order to improve SLiM prediction sensitivity and specificity. QSLiMFinder is designed to identify SLiMs shared between a specific ‘query’ protein (or segment thereof) and a group of proteins that interact with the same PPI partner. QSLiMFinder builds the motif space of putative SLiMs from the query and then searches for enrichment in the remaining proteins. This reduces the motif space and enables the search to be focused on a specific region for which high quality/confidence PPI information is available. For example, such regions could be derived or predicted from solved structures of interacting proteins (Mosca *et al*., 2014; Stein and Aloy, 2010) or binary PPI experiments, such as yeast two-hybrid fragment libraries (Waaijers *et al*., 2013). Although improving all the time, it is questionable whether current PPI data are of sufficient quality and coverage for efficient SLiM discovery (Edwards *et al*., 2012). Therefore, we present a comprehensive benchmark of QSLiMFinder on carefully controlled protein datasets of known SLiMs from ELM (Dinkel *et al*., 2012) and simulated PPI datasets of real human proteins. Results show that QSLiMFinder can predict SLiMs with higher sensitivity than SLiMFinder where specific PPI data are available.

## 2 Algorithm

### 2.1 The SLiMChance algorithm

The SLiMChance statistical model has been described (Edwards *et al*., 2007) and expanded (Davey *et al.*, 2010b) in previous publications but it is useful to highlight key features here before explaining the alterations made by the QSLiMFinder algorithm. SLiMChance uses multiple rounds of the cumulative binomial function, *f*(*k+*;*n*;*p*), which calculates the probability of observing *k* or more successes from *n* independent trials (with replacement), each of which has a probability of success, *p* (Equation 1). When *k* is 1, this simplifies to Equation 2.

(1) $$f\left(k+;n;p\right)=1-\sum_{i=0}^{i<k}\left(\begin{array}{l} n\\ i \end{array}\right)p^{i}{\left(1-p\right)}^{n-i}$$

(2) $$f\left(1+;n;p\right)=1-{\left(1-p\right)}^{n}$$

SLiMChance uses three cycles of the binomial function in which the probability calculated becomes *P* for the next calculation (Table 1). First, confounding evolutionary relationships are removed by grouping proteins through BLAST homology into ‘unrelated protein clusters’ (UPC), such that no protein in one UPC has BLAST-detectable homology (*E* < 1e-4) with a protein in another UPC. For each SLiM, the probability of occurrence in each UPC (as determined by masked amino acid frequencies) is used to calculate the probability of the observed UPC support. The final SLiMChance probability correction for each motif produces the significance estimate *Sig*, which is dependent on the motif search space, *M*. *M* is determined by SLiMBuild parameter settings (Edwards *et al*., 2007), namely the number of defined positions, *L*, and the maximum wildcard spacer length between defined positions, *W* (Equation 3). As such, it is calculated independently for each length, *L.*

(3) $$M=20^{L}{\left(W+1\right)}^{L-1}$$

**Table 1.** Binomial function calculations used in SLiMChance

Score	Probability	*k*	*n*	*P*
p1+	Occurrence of given motif in each unrelated sequence cluster (UPC)	1+	No. sites in UPC	Probability of motif occurrence per site
Prob	Observed (or greater) support in dataset	Observed support(+)	No. UPC	Mean p1+
Sig	Any motif with observed probability (or less)	1+	Motif space (*M*)	Prob

Although SLiMChance is a heuristic estimation of significance (due to the underlying assumptions of independence) it performs very well on both benchmarking data (Edwards *et al*., 2007) and real interaction data (Edwards *et al*., 2012). It has been shown to be a slightly conservative metric, which helps reduce false positives (FPs) but could miss some real motifs as a consequence (Davey *et al*., 2010b; Edwards *et al*., 2007, 2012). (For this reason, the default cut-off for SLiMFinder is 0.1 rather than 0.05.)

### 2.2 Query SLiMFinder motif space correction

QSLiMFinder aims to improve search sensitivity by using prior knowledge concerning one of the motif occurrences to reduce the motif search space, *M* (Table 1). Under this model, a specific ‘Query’ protein (or region thereof) is defined on the basis of external data suggesting that it contains the SLiM of interest. For the ELM LIG_PCNA, for example, PDB (Berman *et al*., 2000) structure 1U76, which features a 15 amino acid peptide of POLD3 interacting with PCNA (Bruning and Shamoo, 2004), could be used to define a query for the PCNA interactome. QSLiMFinder then empirically identifies all motifs within the specified query/region, as constrained by the SLiMBuild parameter settings, to determine *M*. The query is then removed from the search dataset along with any proteins within the same UPC (Supplementary Fig. S1).

QSLiMFinder therefore represents a trade-off as it sacrifices one of the clusters of unrelated proteins (*n*) and an occurrence of the motif (*k*), which increases the (uncorrected) probability of seeing the motif over-represented by chance. In other words, QSLiMFinder observes *k*-1 occurrences in *n*−1 proteins, as opposed to SLiMFinder observing *k* occurrences in *n* proteins. The increase in sensitivity due to reducing the motif space potentially greatly outweighs the deficit produced by removing the query occurrence. For example, SLiMFinder analysis of the human PCNA interactome returned a LIG_PCNA variant, Q.[IL].FF, which was found in 7/74 UPC with a motif space searched (*M*) of 4 320 000 four-position motifs (*L* = 4; *W* = 2; *M* = 20^4^ × 3^3^) (Edwards *et al*., 2012). If POLD3 were used as a query, this would become 6/73 UPC containing the motif but the motif space would be reduced to the 1029 different four-position motifs in POLD3. If the 15 amino acid peptide of POLD3 was used, *M* would be reduced further to only 44 motifs. This represents a reduction in motif space of 3–5 orders of magnitude and a corresponding increase in the significance of over-represented motifs.

## 3 Methods

QSLiMFinder was thoroughly benchmarked on datasets of known motifs and compared with the unmodified SLiMFinder algorithm.

### 3.1 Reduced ELM definitions inferred from known instances

**Fig. 1.**
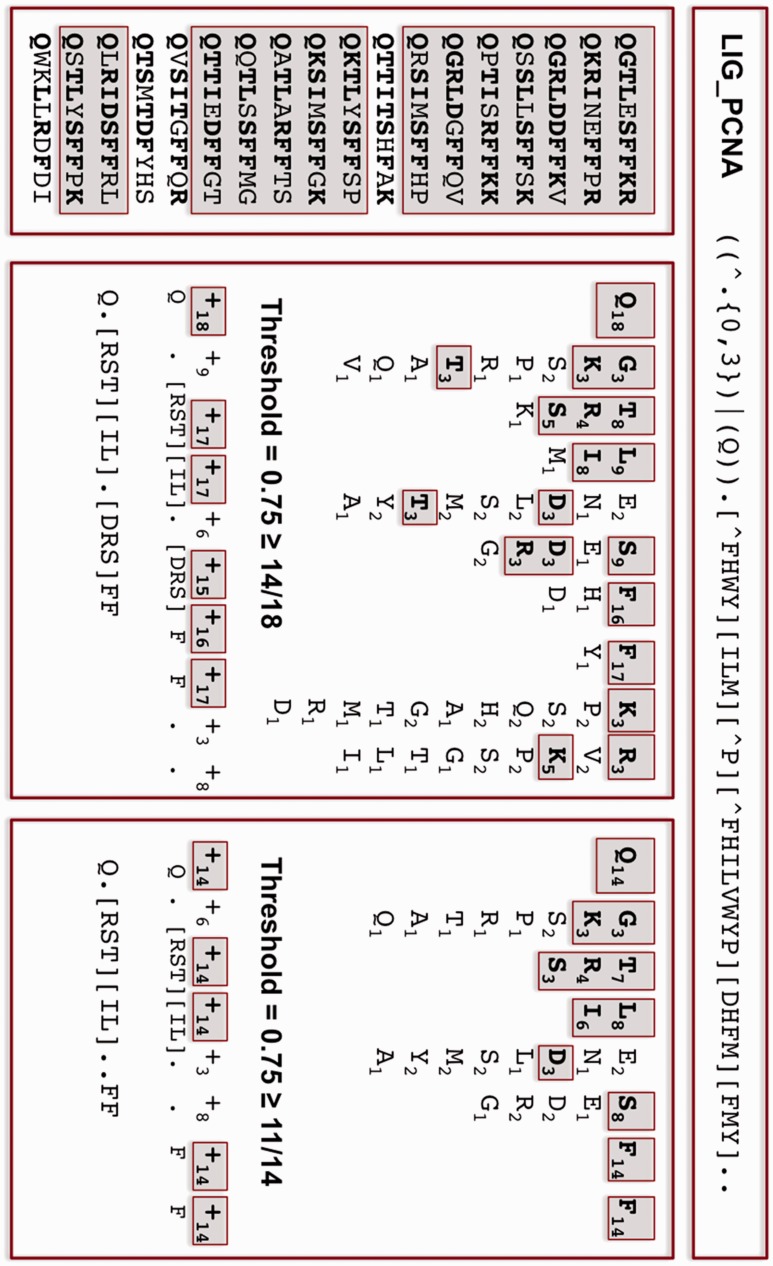
Example reduction of LIG_PCNA motif definition. Each instance of the motif was aligned and used to generate a new motif definition in which only the high frequency recurring residues are included. For each position, amino acids occurring in at least three sequences are identified (bold, highlighted, centre panel). The summed frequency of these amino acids was then calculated and positions with a combined frequency ≥75% were redefined based on these amino acids alone (centre panel). Instances matching the new definition were identified (highlighted, left panel) and the process repeated for this subset (right panel) to produce the final ELM_red_ definition and instances

The ELM database release used in this study (downloaded June 12, 2012) contains over 150 classes of manually annotated eukaryotic SLiMs (Dinkel *et al*., 2012). Because of the manual curation of the motifs, many of the motif definitions incorporate sequence specificity information that is not found in known occurrences of the motif. This information is vital for accurate prediction of novel instances of these ELMs but it presents an unwelcome challenge for *de novo* SLiM prediction benchmarking, as it is impossible for computational tools to achieve the same level of specificity given the lack of information in the input data. In a similar vein, manual curation can include rare variants that prediction methods cannot be expected to recognize. LIG_PCNA, for example, is defined as ((^x{0,3})|(Q))x[^FHWY][ILM][^P][^FHILVWYP][DHFM][FMY]xx where ((^x{0,3})|(Q)) represents ‘glutamine or up to three N-terminal residues’, [^P] represents ‘anything but proline’ and x represents ‘any amino acid’ (Dinkel *et al*., 2012). Each of the non-phenylalanine variants in the last two defined positions, however, occurs in only one LIG_PCNA occurrence in the database (Fig. 1). Complex motif definitions also make it challenging to identify whether a prediction method is returning the correct motif from a given dataset; the more degenerate a regular expression is, the more likely it is to get a match using CompariMotif (Edwards *et al*., 2008) or manual comparisons.

To counter these issues, ELM motifs were redefined purely on the basis of the known occurrences for each motif using SLiMMaker (http://rest.slimsuite.unsw.edu.au/slimmaker). Occurrences were aligned and each position taken in sequence and assessed for a ‘specificity signal’ (Fig. 1):

1. Each individual amino acid variant must occur in at least 3 different occurrences.
2. At least 75% of occurrences must have an amino acid that meets requirement 1, otherwise the position was marked as a wildcard.
3. The maximum number of amino acids for each position was 5. If 6+ different amino acids each occurred in 3+ sequences, the position was marked as a wildcard.

For example, position 3 of the LIG_PCNA motif is defined in ELM as [^FHWY]. Taken together, the 18 LIG_PCNA instances in ELM have the following amino acid composition: 1K, 4R, 5S and 8T. Amino acids R, S and T each comply with 3+ occurrences while K has fewer than three occurrences and is ignored. The summed frequency of R+S+T equals (4+5+8)/18 = 17/18. This exceeds the 0.75 cut-off and therefore position 3 is redefined as [RST], which is a less degenerate version of [^FHWY]. In contrast, position 5 is defined as [^P] and has amino acids: 1A, 3D, 2E, 2L, 2M, 1N, 2S, 3T and 2Y. Although D and T have 3+ occurrences, position 5 is not defined as [DT] because their summed frequency is only (3+3)/18 = 6/18, which does not exceed the 0.75 threshold. Therefore, position 5 is returned as a wildcard.

Leading and trailing wildcards were removed but end of sequence characters for N-terminal (^) and C-terminal ($) positions were included. Original ELM instances that did not match the revised motif were removed and remaining instances subject to another round of SLiMMaker motif definition using the same method. This process was iterated until all retained instances matched the redefined motif. The final ‘reduced’ ELM data are hereon referred to as reduced ELM (ELM_red_) definitions and instances (Supplementary Table S1).

### 3.2 ELM benchmarking data

**Fig. 2.**
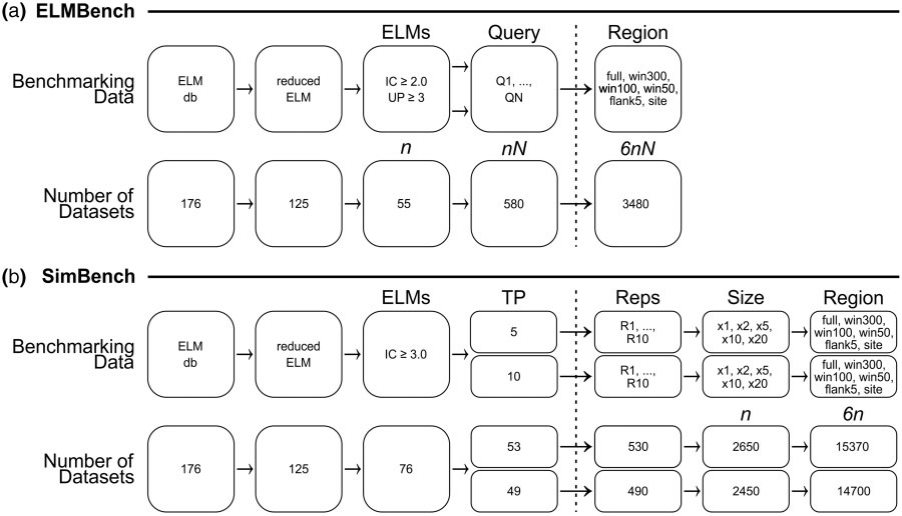
(**a**) ELMBench dataset generation. ELMs are first reduced to only those datasets for which SLiMFinder or QSLiMFinder could theoretically find the ELM_red_ based on the signal within the data (information content of motif and number of unrelated occurrences). For each ELM analysed, each protein is taken in turn and used as a query. Each query is masked at six levels of resolution: (i) Full-length protein; (ii) 300 amino acid window, centred on motif where possible; (iii) 100 amino acid window; (iv) 50 amino acid window; (v) ELM instance plus 2 × 5 amino acid flanking sequences and (vi) ELM instance region only. (**b**) SimBench dataset generation. ELM_red_ definitions with a normalized IC ≥ 3.0 were searched against the human proteome and 10 queries selected (with replacement) to seed 10 replicate datasets. Next, additional ELM_red_-positive proteins were selected at random (without replacement) to make a total of 5 or 10 positive proteins and further human proteins selected at random (without replacement) to make the final simulated datasets of different total sizes (TP×1, ×2, ×5, ×10 and ×20). As with ELMBench, the SimBench queries are masked at same six different levels of site resolution

ELM has been used to benchmark several motif prediction algorithms (Davey *et al*., 2006. 2009, 2010c, 2012b; Edwards *et al*., 2007; Neduva *et al*., 2005). Previous studies have limited benchmarking to ELMs with 3+ unrelated (non-homologous) motif instances. Despite this, some ELMs had too much degeneracy and/or too few instances to be rediscovered, even by a perfect algorithm. Including such datasets in a comparative benchmarking study is pointless as all methods will fail. Therefore, an additional restriction was applied, limiting analysis to ELM_red_ definitions with a normalized information content (Edwards *et al*., 2008) equal or greater than 2.0, an equivalent of having at least two fixed positions. In total, there were 1968 instances belonging to 156 ELM classes, representing 1284 unique proteins. 125 classes (1182 instances) were retained following ELM_red_ redefinition. Of these, 55 had 3+ unrelated motif-containing proteins and were selected for benchmarking, forming the ELM benchmarking (ELMBench) dataset (Fig. 2). To control for possible artefacts due to differences between query proteins, each protein in a given dataset was taken in turn and used as the query (Supplementary Fig. S2).

### 3.3 Simulated and random benchmarking data

A second benchmarking dataset of simulated and random benchmarking data (SimBench) was designed to more accurately reflect the real FP rates of *de novo* SLiM discovery by using random human proteins rather than proteins with known ELM instances. These data consisted of simulated PPI datasets in which a known proportion of any dataset contained a specific ELM motif that interacts with the hypothetical interaction partner of the proteins. This was achieved by first searching a human protein dataset of 23 961 sequences constructed as outlined in Edwards *et al.* (2012) using downloads from December 6, 2012 (Supplementary data). Searches were performed using SLiMProb 1.2 [formerly SLiMSearch 1.x (Davey *et al*., 2010)] and restricted to disordered regions [IUPred (Dosztanyi *et al*., 2005) ≥ 0.2] masked according to relative local conservation (Davey *et al*., 2009; Edwards *et al*., 2007) as described in Edwards *et al.* (2012). The 76 ELM_red_ with a normalized information content (Edwards *et al*., 2008) ≥3.0 were taken in turn to generate 10 replicates of ‘true positive’ (TP) simulated datasets (Fig. 2b). For each dataset, a different query protein was selected (with replacement) from the positive human proteome search results, while the rest of the ‘signal’ proteins (either 5 or 10, including the query) were selected from unrelated proteome hits. Any motif without sufficient unrelated ‘signal’ proteins in the human proteome was excluded. Datasets were completed with ‘noise’ proteins selected at random from the proteome irrespective of whether the motif was found in the protein or not. Five different signal-to-noise ratios were used: 1:0 (‘signal’ only), 1:1, 1:4, 1:9 and 1:19. Each of the simulated datasets was paired with a ‘true negative’ random dataset with the same query protein but in which all other proteins were selected randomly from the proteome. In total, the analysis of each ELM comprised up to 100 pairs of simulated datasets, generated from 10 replicates of 2 different ‘signal’ protein counts and 5 signal-to-noise ratios.

### 3.4 SLiM prediction

SLiM prediction was performed using both SLiMFinder 4.6 and QSLiMFinder 1.7 with default settings. Where disorder masking was applied, residues with an IUPred score <0.2 were masked (Dosztanyi *et al*., 2005), with a minimum (dis)ordered region size of 5 amino acids. Conservation masking used settings and alignments from Edwards *et al.* (2012).

### 3.5 Assessment of SLiM prediction

SLiM predictions were rated as TP, FP or off-target matches (OT). This was achieved by comparing the patterns to the ELM_red_ definitions using CompariMotif 3.8 (Edwards *et al*., 2008). Any CompariMotif hits matching at least two positions with a MatchIC ≥ 1.5 (approximately equivalent to one fixed and one 3-fold degenerate position, or a pair of 2-fold degenerate positions) and a normalized IC ≥ 0.5 (i.e*.* at least half the smallest motif is matched) were classed as motif matches. Motif matches were defined as TP if the ELM matched was the same as (or a variant of) that used to construct the dataset. Remaining motif matches were classed as OT if the pattern had been recognized as a TP in a different dataset, or it matched an ELM with a more stringent criteria of MatchIC ≥ 2.5 or NormIC ≥ 1.0 (e.g*.* the smaller pattern being matched entirely at sites with fixed amino acids or low degeneracy). The remaining patterns were classed as FP.

Once each pattern had been rated, performance metrics were calculated for relevant sets of data:

1. SN, the proportion of datasets returning a TP. (Positive datasets only for SimBench.)
2. The proportion of datasets returning a FP (FPX). (Negative datasets only for SimBench.)

OT motifs were ignored for clarity. Calculating FPX with OT reclassified as TP or FP did not qualitatively affect any of the results presented (data not shown).

For ELMBench, the different numbers of queries for each ELM was normalized by first calculating values for each ELM and then taking the mean values across ELMs. SLiMFinder clusters motifs with overlapping patterns and instances into ‘clouds’. All analysis in this article used only the top-ranked motif in each cloud. Treating each returned pattern independently did not qualitatively affect any of the results presented (data not shown).

### 3.6 Flanking region analysis

To reflect different levels of prior knowledge, six different flanking region strategies were applied to the ELM query sequences (Fig. 2) to reduce the motif space (QSLiMFinder) or sequence search space (SLiMFinder):

1. Full-length proteins (‘none’). This represents the lowest resolution prior data where a specific PPI pair has been identified but the interacting region is totally unknown.
2. 300 amino acid window, centred on the ELM instance (‘win300’). Where the ELM instance was within 150 amino acid of a protein end, the terminal 300 amino acid were used. This represents slightly higher resolution data, e.g*.* where chimera studies or yeast-two-hybrid fragment experiments have narrowed the site of interaction down to a region of a protein.
3. 100 amino acid window, centred on the ELM instance (‘win100’). The terminal 100 amino acid were used if ELM instance was within 50 amino acid of a protein terminus.
4. 50 amino acid window, centred on the ELM instance (‘win50’). The terminal 50 amino acid were used if ELM instance was within 25 amino acid of a protein terminus.
5. Motif instance plus five flanking amino acids in each direction (‘flank5’). This represents a typical SLiM ligand bound to its binding domain where some of the flanking residues are also important for specificity and binding even if they do not contribute to the motif definition itself (Stein and Aloy, 2008).
6. The motif instance only (‘site’). This represents the highest quality prior knowledge, where mutation experiments etc*.* have precisely identified the key region.

### 3.7 Ambiguity in motif definition

SLiMBuild constructs ambiguous positions by combining different fixed SLiM patterns according to an ‘equivalence file’ of permitted ambiguities, provided that they extend dataset coverage (support) versus the individual fixed patterns (Edwards *et al*., 2007). Because QSLiMFinder builds the motif space from the query alone, it cannot incorporate pattern variants found elsewhere in the data without violating the SLiMChance model or inflating the motif space. Therefore, unless otherwise specified, motif ambiguity was switched off for both QSLiMFinder and SLiMFinder, even though the underlying ELM_red_ definitions include ambiguity. Where ambiguity was used, the following sets of equivalencies were used: [ILMVF], [FYW], [FYH], [KRH], [DE], [ST].

## 4 Results

### 4.1 QSLiMFinder increases prediction sensitivity by reducing motif search space

The main aim of QSLiMFinder is to increase the sensitivity of SLiM discovery by using specific ‘query’ data to reduce the motif and sequence search spaces. First, we investigated how well QSLiMFinder returned known motifs from the ELMBench datasets of known SLiM-containing proteins from the ELM database (Dinkel *et al*., 2012). Because ELMs are manually defined and thus contain specificity not necessarily found within the known instances themselves, ELM_red_ definitions were used that should, in principle, be possible to discover (normalized IC ≥ 2.0, 3+ non-homologous occurrences). Queries were restricted to the ELM instance plus five flanking residues on each side and proteins were masked to only include regions predicted disorder (IUpred score ≥ 0.2 [Dosztanyi *et al*., 2005]). Although ELM_red_ definitions could include degenerate positions, which could feature one of several different amino acids, SLiM predictions were restricted to fixed position motifs only. Each ELM-containing protein was selected in turn to be the query and the percentage of datasets returning a match to the known ELM (CompariMotif MatchIC ≥ 1.5, normalized IC ≥ 0.5 [Edwards *et al*., 2008]) calculated for SLiMFinder and QSLiMFinder at different SLiMChance significance levels.

**Fig. 3.**
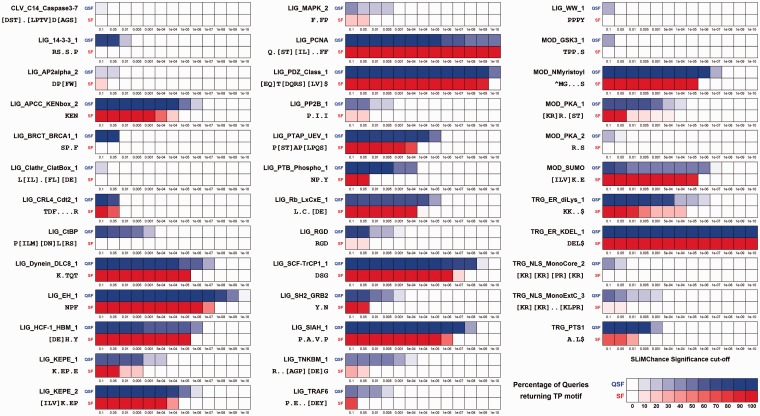
Comparison of QSLiMFinder (QSF, top rows) and SLiMFinder (SF, bottom rows) results for the ELMBench data after searching for true instances of an ELM using a region containing the ELM plus five flanking residues at each side. For each dataset, indicated by its ELM name, the percentage of Queries returning the TP motif at different significance cutoffs is shown. ELM_red_ patterns below each ELM name were used to assess predictions for both QSLiMFinder and SLiMFinder. Fill intensity represents the percentage of queries that return the TP motif according to the scale on the lower right. Disorder masking (IUPred ≥ 0.2) was used for all analysis. ELMs for which neither method returned a TP prediction are not shown

SLiMFinder is known to be conservative (Davey *et al*., 2010b; Edwards *et al*., 2007) and TP results with at least borderline significance (*P* ≤ 0.1) were returned for one or more queries for 28 of the 55 ELM_red_ datasets (Fig. 3). As expected, QSLiMFinder demonstrated greater SN and returned TPs at greater significance for 25 of these ELMs, in addition to returning TPs (*P* ≤ 0.1) for a further nine ELMs. Given its reliance on the query data to generate the motif space, it is not surprising that QSLiMFinder showed greater variability between queries in terms of whether the ELM was returned at a given SLiMChance cut-off. SLiMFinder also demonstrated some query-specific significance, which is likely to result from different variants of ambiguous ELM_red_ motifs in different queries.

**Fig. 4.**
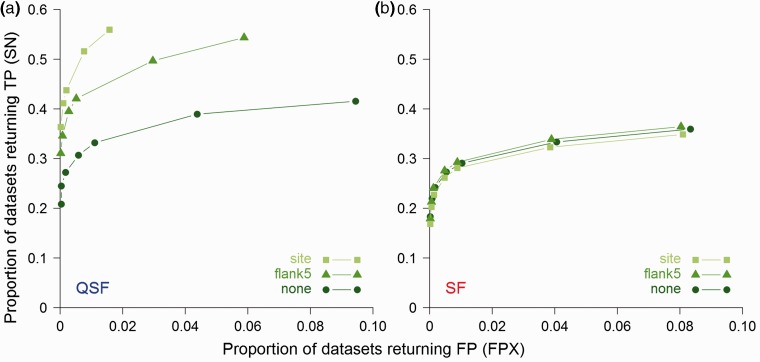
Comparison of (**a**) QSLiMFinder (QSF) and (**b**) SLiMFinder (SF) results on SimBench datasets after searching with fragments of the Query protein of decreasing size. SN, the proportion of datasets returning a TP, is plotted against FPX, the proportion of datasets returning a FP, at different SLiMChance significance cut-offs (0.1, 0.05, 0.01, 0.005, 0.001, 5e-04, 1 e-04). Searches were made with the whole protein (‘none’, circles), with a window of five residues flanking the known ELM at each side (‘flank5’, triangles) or with the region of the motif only (‘site’, squares). For clarity, plots are truncated at the least significant cut-off for which FPX = 0

ELMBench datasets are commonly used for SLiM prediction benchmarking but are quite limited because (i) the number of ELMs is restricted, and (ii) the realism of a dataset in which every protein contains the SLiM is questionable for real world applications. We therefore sought to generate a more extensive benchmarking dataset, SimBench, which would more accurately reflect the nature of real world protein datasets for SLiM prediction and neither rely on, nor be unduly biased by, experimental data. For this, the 76 ELM_red_ patterns with a normalized information content ≥ 3.0 (equivalent of 3+ fixed positions) were used to generate multiple datasets of real human proteins with different numbers of proteins and a range of signal-to-noise ratios, plus a matching number of control datasets of randomly selected human proteins. Again, QSLiMFinder shows greater SN than SLiMFinder, returning TP results for a greater proportion of SimBench datasets (Fig. 4). As expected, the effect is most pronounced when the query region is smallest, as this is when the motif space is most dramatically reduced. For the sake of clarity only those results obtained with the whole protein and the SLiM region with and without flanking residues are displayed, but results with windows of intermediate sizes lie in-between, as expected (data not shown).

### 4.2 QSLiMFinder predictions maintain the high specificity of SLiMFinder

The ability to successfully return known motifs is only one side of a useful SLiM discovery tool. In real life, it is often not known whether a SLiM is present in the data at all, and the statistics granting the ability to successfully avoid the return of FP predictions is critical. (For this reason, we do not benchmark predictions based on ranked scores, which are of limited use in real-world applications of *de novo* SLiM prediction.) Consistent with previous analyses, SLiMFinder is conservative and exhibits high specificity on SimBench, with ∼8% of random datasets returning a significant motif at a relaxed significance threshold of *P* ≤ 0.1 (Fig. 4). Although QSLiMFinder does not have quite the same specificity when the whole query protein is used, the improved SN is not caused by over-prediction and the SLiMChance statistics are still slightly conservative. Reducing the query region increases specificity as well as SN over SLiMFinder, giving a double benefit. This is to be expected as the reduced motif space means that there are fewer patterns that could be over-represented by chance. Although this should be compensated by the reduced multiple testing correction, there are clearly local sequence biases that result in certain patterns being enriched by chance in real proteins (Edwards *et al*., 2012) and reducing the chance of including these in the motif space is likely to have added benefit.

### 4.3 Incorporating ambiguity in QSLiMFinder results in over-prediction

**Fig. 5.**
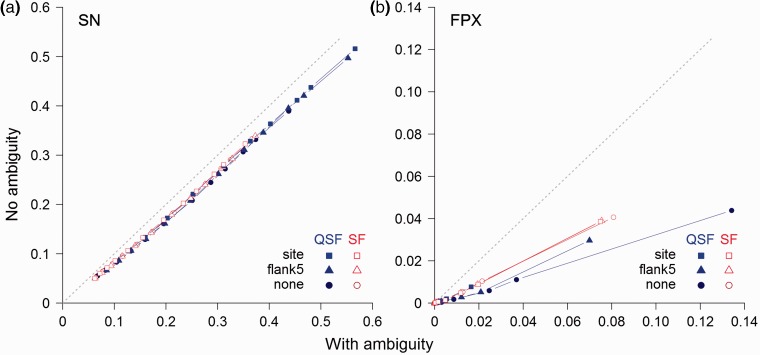
Comparison of the effect of incorporating ambiguity on motif definition on the proportion of SimBench datasets returning (**a**) at least one TP (SN) and (**b**) at least one FP (FPX) when searches are performed using QSLiMFinder (QSF) and SLiMFinder (SF). Results are plot at different SLiMChance significance cut-offs (0.05, 0.01, 0.005, 0.001, 5 e-04, 1 e-04, 1 e-05, 1 e-06, 1 e-07, 1 e-08, 1 e-09, 1 e-10; in panel (**b**) results are truncated at 1 e-04, the least significant cut-off for which FPX = 0.) Searches were made with the whole protein (‘none’, circles), with a window of five residues flanking the known ELM at each side (‘flank5’, triangles) or with the region of the motif only (‘site’, squares)

Reducing the motif space to that of the query does not come without cost. In addition to removing one of the TP instances, the ability to incorporate ambiguity is compromised. SLiMBuild constructs ambiguous positions by combining different fixed SLiM patterns according to an ‘equivalence list’ of permitted ambiguities, provided that they extend dataset coverage (support) versus the individual fixed patterns. Because QSLiMFinder builds the motif space from the query alone, it cannot incorporate pattern variants found elsewhere in the data without violating the SLiMChance model. Incorporating ambiguity in QSLiMFinder therefore results in over-prediction and elevated FP rates, whilst SLiMFinder is less affected (Fig. 5). However, ambiguity can be useful to providing a more nuanced motif definition than fixed position motifs alone (Edwards *et al*., 2007) and does give a marginal improvement in SN (Fig. 5a). A possible workaround is to enable the return of ambiguous motifs but exclude them as FPs unless a significant fixed position pattern is returned in the same motif cloud (set of overlapping motifs [Edwards *et al*., 2007]). This is provided as a new option (cloudfix = T) in SLiMFinder and QSLiMFinder.

### 4.4 Sequence masking can further improve QSLiMFinder sensitivity

It has been previously shown that general sequence masking can improve the sensitivity and specificity of SLiMFinder by reducing the sequence search space (Davey *et al*., 2009; Edwards *et al*., 2007). Therefore, we sought to examine whether additional masking could further boost QSLiMFinder performance by comparing different dataset masking strategies. SLiM prediction was executed with both predicted disorder and relative local conservation masking (‘Bothmask’), disorder masking alone (‘Dismask’) or neither (‘Nomask’). Masking was applied to the entire protein dataset including the query.

**Fig. 6.**
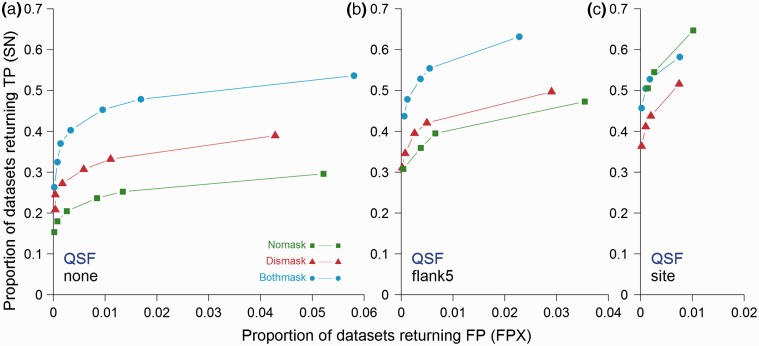
Comparison of QSLiMFinder (QSF) results on SimBench datasets with different masking strategies. The proportion of datasets returning a true motif (SN) is plotted against the proportion of datasets returning a false hit (FPX) for average values of controlled signal-noise combinations at each different SLiMChance significance cut-off (0.05, 0.01, 0.005, 0.001, 5 e-04, 1 e-04, 5 e-05). Searches were made (**a**) without further masking of the query (‘Nomask’, squares), (**b**) masking out disordered regions (‘Dismask’, triangles) or (**c**) masking out both disordered and evolutionary conserved positions (‘Bothmask’, circles). Results were obtained with (**a**) the whole protein as the query, (**b**) with a window of five residues at each side of the known motif or (**c**) with the motif only. For clarity, plots are truncated at the least significant cut-off for which FPX = 0

In general, reducing the sequence space through sequence masking added to the query region benefits for QSLiMFinder SN (Fig. 6). This is to be expected, as additional masking of the query will further reduce the motif space, whilst overall masking of the dataset will reduce the sequence space. The FP rate was also improved, albeit by a smaller magnitude. The exception was for the site-specific query region masking, for which the Nomask strategy was most successful (Fig. 6). This is because it is quite rare to return the precise motif being sought and many TP matches incorporate an additional flanking or internal residue that is over-represented but not part of the formal motif definition. This is particularly true when fixed position variants of ambiguous motifs are being sought, as in these analyses. Extremely stringent masking will eliminate the possibility of such extended patterns being returned. For this reason, unless the user is extremely confident about the precise location and context of a SLiM, it is probably a good idea to include some flanking sequence. In real data, the utility of masking is not so clear-cut as it cannot be guaranteed that the SLiM occurrences being sought meet the masking criteria. However, where there is confidence that the criteria are met, it can make a big difference. In other scenarios, using QSLiMFinder with precise location data for the query can reduce the need for additional sequence masking.

### 4.5 Prediction accuracy is highly dependent on the signal-to-noise ratio of the data

**Fig. 7.**
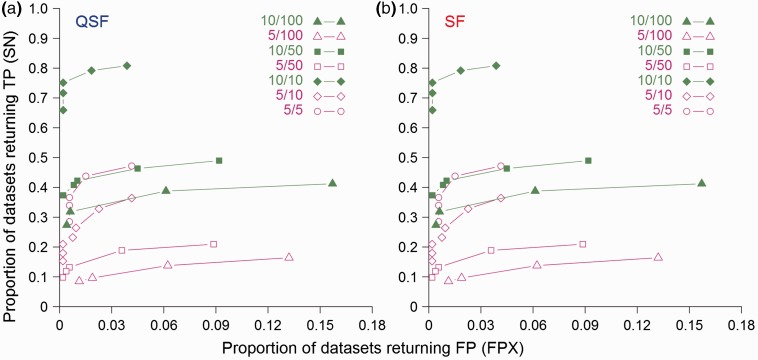
Comparison of (**a**) QSLiMFinder (QSF) and (**b**) SLiMFinder (SF) results on SimBench datasets with different signal-to-noise ratios. The proportion of datasets returning a true motif (SN) is plotted against the proportion of datasets returning a false hit (FPX) at each different SLiMChance significance cut-off (0.1, 0.05, 0.01, 0.005, 0.001, 5 e-04, 1 e-04). Selected combinations of signal (5, open symbols; 10, filled symbols) and dataset sizes (5, circles; 10, diamonds; 50, squares; 100, triangles) are displayed. Searches were made using the whole protein with disorder masking. For clarity, plots are truncated at the least significant cut-off for which FPX = 0

Real protein datasets vary wildly in terms of the number of proteins they contain (Edwards *et al*., 2012). In general, an unknown fraction of these proteins will contain the SLiM being sought. The remaining proteins are ‘noise’, which interact with the target protein via a different mechanism. The SimBench data were generated with two different TP counts (5 or 10 per dataset) and five different signal-to-noise ratios to investigate the effects of data quality and quantity. As expected, the composition of the dataset is highly relevant to determine the trade-off between sensitivity and specificity. Intuitively, increasing the signal-to-noise ratio improves the sensitivity of prediction for both SLiMFinder and QSLiMFinder (Fig. 7). At equal signal-to-noise ratios, larger datasets also give a marked increase in true motifs, indicating that the SLiMChance over-representation statistics become more sensitive as the number of occurrences increases, which is not surprising given its foundation on the binomial distribution. However, in line with previous results, increasing the dataset size also increases the likelihood of a FP being returned (Edwards *et al*., 2007, 2012). This is most likely due to the effects of small local biases in amino acid composition being amplified as dataset sizes increase.

## 5 Discussion

Query SLiMFinder (QSLiMFinder) is a modified version of SLiMFinder that makes use of a specific query protein (or region thereof) to reduce the motif search space. By reducing the corresponding multiple testing correction, QSLiMFinder can increase the sensitivity of *de novo* SLiM prediction (Fig. 4). By reducing the number of motifs that could be susceptible to sequence biases within the data, QSLiMFinder also reduces the number of datasets returning FP predictions (Fig. 4). Intuitively, the more precisely the query sequence can be restricted to the site of the interaction, the smaller the motif space is and the larger the benefit provided by QSLiMFinder. Furthermore, the explicit use of a specific PPI pair will make subsequent interpretation and validation easier.

Despite these benefits, there are scenarios in which SLiMFinder remains the more appropriate choice, even when specific PPI data are available. QSLiMFinder reduces the motif space by sacrificing an occurrence of the motif. For small datasets, SLiMFinder is more likely to cope with the limited number of motif occurrences that will challenge the sensitivity of SLiMChance. Furthermore, QSLiMFinder cannot handle ambiguity as well as SLiMFinder (Fig. 5). Because the benefits of QSLiMFinder are small when full-length queries are used, it might be more appropriate to use SLiMFinder in these cases unless the query protein is itself very short. Overall, the results of our analysis point to different applications for SLiMFinder and QSLiMFinder, with the latter best-suited to exploit specific information about interaction sites.

In this article, we also introduce SLiMBench, a combination of carefully formulated benchmarking datasets and a rule-based automated benchmarking tool for consistent, repeatable comparison of *de novo* SLiM prediction methods. The design and scale of these data have provided additional insights regarding dataset design with respect to signal-to-noise. Prediction SN (TP rate) is primarily influenced by the number of proteins in the dataset containing the motif, whereas specificity (FP rate) is predominantly influenced by overall dataset size (Fig. 7). Due to the stringency of the SLiMChance statistics underpinning SLiMFinder and QSLiMFinder, both programs are more tolerant of increased noise than reduced signal, consistent with previous results (Edwards *et al*., 2007, 2012). Therefore, an interesting dilemma may arise when building a new search dataset, between seeking a better signal-to-noise ratio to enhance sensitivity and increasing dataset size for extended motif coverage. Maximizing the signal-to-noise ratio of protein datasets will hopefully maximize the accuracy of predictions but extra caution should be taken when removing unfavourable proteins and/or masking sequences, lest motif instances are accidentally removed. On the other hand, if high precision (i.e*.* a low FP rate) is critical, bloating the dataset with uninteresting sequences should be avoided. The next step will be to apply these principles to real PPI data.

## Supplementary Material

Supplementary Data

## References

[btv155-B1] Babu M.M. (2011) Intrinsically disordered proteins: regulation and disease. Curr. Opin. Struct. Biol., 21, 432–440. 21514144 10.1016/j.sbi.2011.03.011

[btv155-B2] Berman H.M. (2000) The Protein Data Bank. Nucleic Acids Res., 28, 235–242. 10592235 10.1093/nar/28.1.235 PMC102472

[btv155-B3] Bruning J.B. Shamoo Y. (2004) Structural and thermodynamic analysis of human PCNA with peptides derived from DNA polymerase-delta p66 subunit and flap endonuclease-1. Structure, 12, 2209–2219. 15576034 10.1016/j.str.2004.09.018

[btv155-B4] Davey N.E.*.* (2006) SLiMDisc: short, linear motif discovery, correcting for common evolutionary descent. Nucleic Acids Res., 34, 3546–3554. 16855291 10.1093/nar/gkl486 PMC1524906

[btv155-B5] Davey N.E.*.* (2009) Masking residues using context-specific evolutionary conservation significantly improves short linear motif discovery. Bioinformatics, 25, 443–450. 19136552 10.1093/bioinformatics/btn664

[btv155-B6] Davey N.E. (2010a) Computational identification and analysis of protein short linear motifs. Front. Biosci., 15, 801–825. 10.2741/3647 20515727

[btv155-B7] Davey N.E.*.* (2010b) Estimation and efficient computation of the true probability of recurrence of short linear protein sequence motifs in unrelated proteins. BMC Bioinformatics, 11, 14. 20055997 10.1186/1471-2105-11-14 PMC2819990

[btv155-B8] Davey N.E. (2010c) SLiMFinder: a web server to find novel, significantly over-represented, short protein motifs. Nucleic Acids Res., 38(Web Server issue), W534–W539. 20497999 10.1093/nar/gkq440 PMC2896084

[btv155-B9] Davey N.E. (2010d) SLiMSearch: a webserver for finding novel occurrences of short linear motifs in proteins, incorporating sequence context. Lect Notes Bioinform., 6282, 50–61.

[btv155-B10] Davey N.E. (2011) How viruses hijack cell regulation. Trends Biochem. Sci., 36, 159–169. 21146412 10.1016/j.tibs.2010.10.002

[btv155-B11] Davey N.E. (2012a) Attributes of short linear motifs. Mol. Biosyst., 8, 268–281. 21909575 10.1039/c1mb05231d

[btv155-B12] Davey N.E. (2012b) SLiMPrints: conservation-based discovery of functional motif fingerprints in intrinsically disordered protein regions. Nucleic Acids Res., 40, 10628–10641. 22977176 10.1093/nar/gks854 PMC3510515

[btv155-B13] Diella F. (2008) Understanding eukaryotic linear motifs and their role in cell signaling and regulation. Front. Biosci., 13, 6580–6603. 18508681 10.2741/3175

[btv155-B14] Dinkel H. (2012) ELM—the database of eukaryotic linear motifs. Nucleic Acids Res., 40(Database issue), D242–D251. 22110040 10.1093/nar/gkr1064 PMC3245074

[btv155-B15] Dinkel H. (2014) The eukaryotic linear motif resource ELM: 10 years and counting. Nucleic Acids Res., 42, D259–D266. 24214962 10.1093/nar/gkt1047 PMC3964949

[btv155-B16] Dosztanyi Z. (2005) IUPred: web server for the prediction of intrinsically unstructured regions of proteins based on estimated energy content. Bioinformatics, 21, 3433–3434. 15955779 10.1093/bioinformatics/bti541

[btv155-B17] Edwards R.J. Palopoli N. (2015) Computational prediction of short linear motifs from protein sequences. Methods Mol. Biol., 1268, 89–141. 25555723 10.1007/978-1-4939-2285-7_6

[btv155-B18] Edwards R.J. (2007) SLiMFinder: a probabilistic method for identifying over-represented, convergently evolved, short linear motifs in proteins. PLoS One, 2, e967. 17912346 10.1371/journal.pone.0000967 PMC1989135

[btv155-B19] Edwards R.J. (2008) CompariMotif: quick and easy comparisons of sequence motifs. Bioinformatics, 24, 1307–1309. 18375965 10.1093/bioinformatics/btn105

[btv155-B20] Edwards R.J. (2012) Interactome-wide prediction of short, disordered protein interaction motifs in humans. Mol. Biosyst., 8, 282–295. 21879107 10.1039/c1mb05212h

[btv155-B21] Lieber D.S. (2010) Large-scale discovery and characterization of protein regulatory motifs in eukaryotes. PLoS One, 5, e14444. 21206902 10.1371/journal.pone.0014444 PMC3012054

[btv155-B22] Mosca R (2014) 3did: a catalog of domain-based interactions of known three-dimensional structure. Nucleic Acids Res., 42, D374–D379. 24081580 10.1093/nar/gkt887 PMC3965002

[btv155-B23] Neduva V. Russell R.B. (2005) Linear motifs: evolutionary interaction switches. FEBS Lett., 579, 3342–3345. 15943979 10.1016/j.febslet.2005.04.005

[btv155-B24] Neduva V. Russell R.B. (2006) Peptides mediating interaction networks: new leads at last. Curr. Opin. Biotechnol., 17, 465–471. 16962311 10.1016/j.copbio.2006.08.002

[btv155-B25] Neduva V. (2005) Systematic discovery of new recognition peptides mediating protein interaction networks. PLoS Biol., 3, e405. 16279839 10.1371/journal.pbio.0030405 PMC1283537

[btv155-B26] Pancsa R. Fuxreiter M. (2012) Interactions via intrinsically disordered regions: what kind of motifs? IUBMB Life, 64, 513–520. 22535488 10.1002/iub.1034

[btv155-B27] Russell R.B. Gibson T.J. (2008) A careful disorderliness in the proteome: sites for interaction and targets for future therapies. FEBS Lett., 582, 1271–1275. 18284921 10.1016/j.febslet.2008.02.027

[btv155-B28] Stein A. Aloy P. (2008) Contextual specificity in peptide-mediated protein interactions. PLoS One, 3, e2524. 18596940 10.1371/journal.pone.0002524 PMC2438476

[btv155-B29] Stein A. Aloy P. (2010) Novel peptide-mediated interactions derived from high-resolution 3-dimensional structures. PLoS Comput. Biol., 6, e1000789. 20502673 10.1371/journal.pcbi.1000789 PMC2873903

[btv155-B30] Tompa P. (2011) Unstructural biology coming of age. Curr. Opin. Struct. Biol., 21, 419–425. 21514142 10.1016/j.sbi.2011.03.012

[btv155-B31] Tuncbag N. (2009) Towards inferring time dimensionality in protein-protein interaction networks by integrating structures: the p53 example. Mol. Biosyst., 5, 1770–1778. 19585003 10.1039/b905661k PMC2898629

[btv155-B32] Waaijers S. (2013) Identification of human protein interaction domains using an ORFeome-based yeast two-hybrid fragment library. J. Proteome Res., 12, 3181–3192. 23718855 10.1021/pr400047p

