# Supplementary material for: QSLiMFinder: improved short linear motif prediction using specific query protein data
Source: Bioinformatics. 2015 Mar 19;31(14):2284–93. doi: 10.1093/bioinformatics/btv155 (PMC4495300; doi:10.1093/bioinformatics/btv155)
Supplement: Supplementary Data [file supp_31_14_2284__index.html]

QSLiMFinder: improved short linear motif prediction using specific query protein data — QSLiMFinder: improved short linear motif prediction using specific query protein data — QSLiMFinder: improved short linear motif prediction using specific query protein data — Supplementary Data 

# QSLiMFinder: improved short linear motif prediction using specific query protein data

## Supplementary Data

files

**Files in this Data Supplement:**

- Supplementary Data - docx file
