# Supplementary material for: QSLiMFinder: improved short linear motif prediction using specific query protein data
Source: Bioinformatics. 2015 Mar 19;31(14):2284–93. doi: 10.1093/bioinformatics/btv155 (PMC4495300; doi:10.1093/bioinformatics/btv155)
Supplement: Supplementary Data [file supp_btv155_BIOINF-2014-1232.Revised.SI.docx]

SUPPLEMENTARY DATA for PALOPOLI ET AL. - QSLiMFinder: improved short linear motif prediction using specific query protein data

Supplementary Table 1. SLiMMaker ELM_red_ motif definitions.

| ELM Motif | ELM Definition | N_ELM_^a^ | Reduced ELM_red_ SLiMMaker Definition | N_red_^a^ |
| --- | --- | --- | --- | --- |
| CLV_C14_Caspase3-7 | [DSTE][^P][^DEWHFYC]D[GSAN] | 36 | [DST].[LPTV]D[AGS] | 22 |
| CLV_MEL_PAP_1 | [ILV]..[R][VF][GS]. | 12 | NRFG | 7 |
| CLV_PCSK_FUR_1 | R.[RK]R. | 12 | R.[KR]R | 12 |
| LIG_14-3-3_1 | R.[^P]([ST])[^P]P | 7 | RS.S.P | 6 |
| LIG_14-3-3_2 | R..[^P]([ST])[IVLM]. | 6 | R...S | 6 |
| LIG_14-3-3_3 | [RHK][STALV].([ST]).[PESRDIFTQ] | 15 | R..S | 10 |
| LIG_AGCK_PIF_1 | F..[FWY][ST][FY] | 9 | F.GF.Y | 5 |
| LIG_AGCK_PIF_2 | F..[FWY][DE][FY] | 5 | FEGFEY | 3 |
| LIG_AGCK_PIF_3 | F..F$ | 5 | F..F$ | 5 |
| LIG_AP2alpha_1 | F.D.F | 11 | F[EG]D.F | 10 |
| LIG_AP2alpha_2 | DP[FW] | 50 | DP[FW] | 50 |
| LIG_APCC_Dbox_1 | .R..L..[LIVM]. | 9 | R..L..[IV] | 7 |
| LIG_APCC_KENbox_2 | .KEN. | 13 | KEN | 13 |
| LIG_AP_GAE_1 | [DE][DES][DEGAS]F[SGAD][DEAP][LVIMFD] | 11 | DD.F..F | 7 |
| LIG_Actin_RPEL_3 | [IL]..[^P][^P][^P][^P]R.....[IL]..[^P][^P][ILV][ILM] | 13 | LNEKIA.RPGP.ELVEKNIL | 3 |
| LIG_Actin_WH2_2 | [^R]..((.[ILMVF])\|([ILMVF].))[^P][^P][ILVM].{4,7}L(([KR].)\|(NK))[VATIGS] | 13 | I | 5 |
| LIG_BIR_III_2 | DA.P. | 3 | DA.P | 3 |
| LIG_BIR_III_3 | ^M{0,1}A.[AP]. | 4 | ^MA..F | 3 |
| LIG_BRCT_BRCA1_1 | .(S)..F | 5 | SP.F | 5 |
| LIG_COP1 | [DE][DE]...VP[DE] | 4 | EE...VP | 3 |
| LIG_CORNRBOX | L[^P]{2,2}[HI]I[^P]{2,2}[IAV][IL] | 4 | L...I | 4 |
| LIG_CRL4_Cdt2_1 | [NQ]{0,1}..[ILMV]T[DEN][FY][FY].{2,3}[KR]{2,3}[^DE] | 5 | TDF....R | 3 |
| LIG_CYCLIN_1 | [RK].L.{0,1}[FYLIVMP] | 24 | [KR].L | 24 |
| LIG_Clathr_ClatBox_1 | L[IVLMF].[IVLMF][DE] | 18 | L[IL].[FL][DE] | 15 |
| LIG_CtBP | [PG][LVIPME][DENS]L[VASTRGE] | 33 | P[ILM][DN]L[RS] | 19 |
| LIG_Dynein_DLC8_1 | [^P].[KR].TQT | 8 | K.TQT | 7 |
| LIG_EH_1 | .NPF. | 85 | NPF | 85 |
| LIG_EVH1_1 | [FILVY].{0,1}P.[PAILSK]P | 20 | FPPPP | 10 |
| LIG_EVH1_2 | PP..F | 8 | PP..F | 8 |
| LIG_EVH1_3 | [FY].[FW].....[LMVIF]P.P[DE] | 3 | FH...D.P.P | 3 |
| LIG_FAT_LD_1 | [LV][DE][^P][LM][LM][^P][^P]L[^P] | 3 | LD.L...L | 3 |
| LIG_FHA_1 | ..(T)..[ILV]. | 4 | T.E | 3 |
| LIG_FHA_2 | ..(T)..[DE]. | 6 | T..[DE] | 6 |
| LIG_GLEBS_BUB3_1 | [EN][FYLW][NSQ].EE[ILMVF][^P][LIVMFA] | 5 | EFS.EE..A | 3 |
| LIG_HCF-1_HBM_1 | [DE]H.Y | 17 | [DE]H.Y | 17 |
| LIG_HOMEOBOX | [FY][DEP]WM | 16 | [FY]PWM | 13 |
| LIG_HP1_1 | P[MVLIRWY]V[MVLIAS][LM] | 8 | P.V.L | 7 |
| LIG_IQ | ...[SACLIVTM]..[ILVMFCT]Q.{3,3}[RK].{4,5}[RKQ].. | 39 | [KVY].[AV].[KLV]IQ[AKRS].[FY]R[AGKR][FHY][KL][AKL][KR][KR] | 16 |
| LIG_KEPE_1 | [VILMFT]K.EP.[DE] | 5 | K.EP.E | 5 |
| LIG_KEPE_2 | [VILMFT]K.EP.{2,3}[DE] | 12 | [ILV]K.EP | 12 |
| LIG_KEPE_3 | [VILMFT]K.EP....[DE] | 4 | K.EP....E | 3 |
| LIG_LYPXL_L_2 | [LM]YP...[LI][^P][^P][LI] | 3 | LYP.TSL.SL | 3 |
| LIG_LYPXL_S_1 | [LM]YP.[LI] | 13 | LYP.L | 11 |
| LIG_MAD2 | [KR][IV][LV].....P | 6 | IL.L...P | 3 |
| LIG_MAPK_1 | [KR]{0,2}[KR].{0,2}[KR].{2,4}[ILVM].[ILVF] | 15 | [KR] | 15 |
| LIG_MAPK_2 | F.FP | 7 | F.FP | 5 |
| LIG_MDM2 | F...W..[LIV] | 3 | F...W..L | 3 |
| LIG_NRBOX | [^P]L[^P][^P]LL[^P] | 23 | L..LL | 23 |
| LIG_OCRL_FandH_1 | .F[^P][^P][KRIL]H[^P][^P][YLMFH][^P]... | 3 | F...H | 3 |
| LIG_ODPH_VHL_1 | [IL]A(P).{6,8}[FLIVM].[FLIVM] | 8 | LAP | 6 |
| LIG_PAM2_1 | ..[LFP][NS][PIVTAFL].A..(([FY].[PYLF])\|(W..)). | 22 | S.LNP.A..F.P | 5 |
| LIG_PCNA | ((^.{0,3})\|(Q)).[^FHWY][ILM][^P][^FHILVWYP][DHFM][FMY].. | 19 | Q.[ST][IL]..FF | 10 |
| LIG_PDZ_Class_1 | ...[ST].[ACVILF]$ | 32 | [EQ]T[DQRS][LV]$ | 12 |
| LIG_PIKK_1 | [DEN][DEN].{2,3}[ILMVA][DEN][DEN]L | 4 | EE....DL | 3 |
| LIG_PP1 | ..[RK].{0,1}[VIL][^P][FW]. | 17 | [KR]..F | 14 |
| LIG_PP2B_1 | .P[^P]I[^P][IV][^P] | 7 | P.I.I | 6 |
| LIG_PTAP_UEV_1 | .P[TS]AP. | 25 | P[ST]AP[LPQS] | 20 |
| LIG_PTB_Apo_2 | (.[^P].NP.[FY].)\|(.[ILVMFY].N..[FY].) | 19 | [GN][FY].NP[TV]Y | 6 |
| LIG_PTB_Phospho_1 | (.[^P].NP.(Y))\|(.[ILVMFY].N..(Y)) | 16 | NP.Y | 12 |
| LIG_RAPTOR_TOS_1 | F[EDQS][MILV][ED][MILV]((.{0,1}[ED])\|($)) | 5 | F..D | 5 |
| LIG_RGD | RGD | 21 | RGD | 21 |
| LIG_RRM_PRI_1 | .[ILVM]LG..P. | 3 | LG..P | 3 |
| LIG_Rb_LxCxE_1 | [LI].C.[DE] | 32 | L.C.[DE] | 31 |
| LIG_Rb_pABgroove_1 | ..[LIMV]..[LM][FY]D. | 3 | L.D | 3 |
| LIG_SCF-TrCP1_1 | D(S)G.{2,3}([ST]) | 15 | DSG | 15 |
| LIG_SCF_FBW7_1 | [LIVMP].{0,2}(T)P..([ST]) | 5 | P | 5 |
| LIG_SCF_Skp2-Cks1_1 | ..[DE].(T)P.K | 3 | VEQTP.K | 3 |
| LIG_SH2_GRB2 | (Y).N. | 14 | Y.N | 14 |
| LIG_SH2_SRC | (Y)[QDEVAIL][DENPYHI][IPVGAHS] | 16 | Y..[IPV] | 12 |
| LIG_SH2_STAT3 | (Y)..Q | 9 | Y..Q | 9 |
| LIG_SH2_STAT5 | (Y)[VLTFIC].. | 12 | Y | 12 |
| LIG_SH3_1 | [RKY]..P..P | 5 | P..P | 5 |
| LIG_SH3_2 | P..P.[KR] | 13 | P.[PV]P.R | 6 |
| LIG_SH3_3 | ...[PV]..P | 10 | P..P | 8 |
| LIG_SIAH_1 | .P.A.V.P[^P] | 9 | P.A.V.P | 9 |
| LIG_SPAK-OSR1_1 | RF[^P][IV]. | 13 | RF.V[ST] | 8 |
| LIG_Sin3_1 | [LIV]..[LM]L.AA.[FY][LI] | 4 | LLEAA..L | 4 |
| LIG_Sin3_2 | [FHYM].A[AV].[VAC]L[MV].[MI] | 3 | A.E.L..M | 3 |
| LIG_SxIP_EBH_1 | ([KR][^ED]{0,5}[ST].IP[^ED]{5,5})\|([^ED]{5,5}[ST].IP[^ED]{0,5}[KR]) | 9 | [PS] | 9 |
| LIG_TNKBM_1 | .R..[PGAV][DEIP]G. | 17 | R..[AGP][DE]G | 15 |
| LIG_TPR | EEVD$ | 9 | EEVD$ | 9 |
| LIG_TRAF2_1 | [PSAT].[QE]E | 14 | [PST].[EQ]E | 13 |
| LIG_TRAF6 | ..P.E..[FYWHDE]. | 20 | P.E..[DEY] | 19 |
| LIG_TRFH_1 | [FY].L.P | 3 | L.P | 3 |
| LIG_USP7_1 | [PA][^P][^FYWIL]S[^P] | 4 | S | 4 |
| LIG_WRPW_1 | [WFY]RP[WFY].{0,7}$ | 95 | [WY]RP[WY] | 93 |
| LIG_WW_1 | PP.Y | 17 | PPPY | 13 |
| LIG_WW_Pin1_4 | ...([ST])P. | 84 | [ST]P | 84 |
| LIG_eIF4E_1 | Y....L[VILMF] | 13 | Y....LL | 10 |
| MOD_CDK_1 | ...([ST])P.[KR] | 5 | SP.K | 3 |
| MOD_CK2_1 | ...([ST])..E | 5 | A.S..E | 3 |
| MOD_CMANNOS | (W)..W | 24 | W[GS][EPS]W | 12 |
| MOD_GSK3_1 | ...([ST])...[ST] | 5 | TPP.S | 3 |
| MOD_N-GLC_1 | .(N)[^P][ST].. | 149 | N.[ST] | 149 |
| MOD_N-GLC_2 | (N)[^P]C | 5 | N.C | 5 |
| MOD_NMyristoyl | ^M{0,1}(G)[^EDRKHPFYW]..[STAGCN][^P] | 13 | ^MG...S | 11 |
| MOD_OFUCOSY | C.{3,5}([ST])C | 4 | C.NGGTC | 3 |
| MOD_PIKK_1 | ...([ST])Q.. | 27 | [ST]Q | 27 |
| MOD_PKA_1 | [RK][RK].([ST])[^P].. | 23 | [KR]R.[ST] | 22 |
| MOD_PKA_2 | .R.([ST])[^P].. | 25 | R.S | 25 |
| MOD_PKB_1 | R.R..([ST])[^P].. | 4 | R.R..S | 4 |
| MOD_ProDKin_1 | ...([ST])P.. | 32 | [ST]P | 32 |
| MOD_SPalmitoyl_4 | ^M{0,1}G(C)..S[AKS] | 5 | ^MGC..S | 5 |
| MOD_SUMO | [VILMAFP](K).E | 38 | [ILV]K.E | 34 |
| MOD_TYR_CSK | [TAD][EA].Q(Y)[QE].[GQA][PEDLS] | 12 | TE[GP]QYQ[PQ][GQ][EP] | 8 |
| MOD_TYR_DYR | ..[RKTC][IVL]Y[TQHS](Y)[IL]QSR | 9 | GQRIYQYIQSR | 5 |
| MOD_TYR_ITIM | [ILV].(Y)..[ILV] | 4 | ITY..L | 3 |
| MOD_TYR_ITSM | ..T.(Y)..[IV] | 11 | T.Y..[IV] | 11 |
| TRG_AP2beta_CARGO_1 | [DE].{1,2}F[^P][^P][FL][^P][^P][^P]R | 4 | D | 4 |
| TRG_ENDOCYTIC_2 | Y..[LMVIF] | 13 | Y..[IL] | 10 |
| TRG_ER_FFAT_1 | [DE].{0,4}E[FY][FYK]D[AC].[ESTD] | 20 | D | 5 |
| TRG_ER_KDEL_1 | [KRHQSAP][DENQT]EL$ | 12 | DEL$ | 11 |
| TRG_ER_diArg_1 | ([LIVMFYWPR]R[^YFWDE]{0,1}R)\|(R[^YFWDE]{0,1}R[LIVMFYWPR]) | 26 | [LR]R | 20 |
| TRG_ER_diLys_1 | K.{0,1}K.{2,3}$ | 13 | KK..$ | 8 |
| TRG_Golgi_diPhe_1 | Q.{6,6}FF.{6,7}$ | 11 | Q..YL.RFF | 5 |
| TRG_LysEnd_APsAcLL_1 | [DERQ]...L[LVI] | 14 | [DE]...LL | 9 |
| TRG_LysEnd_APsAcLL_3 | [DET]E[RK].PL[LI] | 5 | L | 5 |
| TRG_LysEnd_GGAAcLL_1 | D..LL.{1,2}$ | 6 | D..LL..$ | 5 |
| TRG_LysEnd_GGAAcLL_2 | S[LW]LD[DE]EL[LM] | 4 | SLLD.EL | 3 |
| TRG_NES_CRM1_1 | ([DEQ].{0,1}[LIM].{2,3}[LIVMF][^P]{2,3}[LMVF].[LMIV].{0,3}[DE])\|([DE].{0,1}[LIM].{2,3}[LIVMF][^P]{2,3}[LMVF].[LMIV].{0,3}[DEQ]) | 16 | [DEQ] | 16 |
| TRG_NLS_Bipartite_1 | [KR][KR].{7,15}[^DE]((K[RK])\|(RK))(([^DE][KR])\|([KR][^DE]))[^DE] | 8 | KR | 8 |
| TRG_NLS_MonoCore_2 | [^DE]((K[RK])\|(RK))[KRP][KR][^DE] | 14 | [KR][KR][PR][KR] | 13 |
| TRG_NLS_MonoExtC_3 | [^DE]((K[RK])\|(RK))(([^DE][KR])\|([KR][^DE]))(([PKR])\|([^DE][DE])) | 15 | [KR][KR]..[KLPR] | 15 |
| TRG_NLS_MonoExtN_4 | (([PKR].{0,1}[^DE])\|([PKR]))((K[RK])\|(RK))(([^DE][KR])\|([KR][^DE]))[^DE] | 17 | [KPR].[KR].[KR] | 13 |
| TRG_PTS1 | (.[SAPTC][KRH][LMFI]$)\|([KRH][SAPTC][NTS][LMFI]$) | 5 | A.L$ | 3 |

a. Number of instances matching ELM (N_ELM_) and ELM_red_ definitions.

Supplementary Table 2. Top ranked motifs from ELMBench returned by QSLiMFinder and SLiMFinder, restricting the search to disordered regions and building the motif space on the ELM instance plus five flanking residues at each side.

| ELM Motif | **ELM_red_** | **QSLiMFinder** | | | **SLiMFinder** | | |
| --- | --- | --- | --- | --- | --- | --- | --- |
|  |  | **Pattern^a^** | **Sig^b^** | **Support^c^** | **Pattern^a^** | **Sig^b^** | **Support^c^** |
| CLV_C14_Caspase3-7 | [DST].[LPTV]D[AGS] | - | 0.035 | 8/17 (8/9) | - | 0.1 | 0/17 (0/9) |
| CLV_PCSK_FUR_1 | R.[KR]R | - | 0.046 | 4/11 (4/9) | - | 0.1 | 0/11 (0/9) |
| LIG_14-3-3_1 | RS.S.P | RS.S.P | 0.009 | 5/5 (4/4) | - | 0.1 | 0/5 (0/4) |
| LIG_14-3-3_2 | R...S | - | 0.089 | 6/6 (6/6) | - | 0.1 | 0/6 (0/6) |
| LIG_14-3-3_3 | R..S | - | 0.003 | 7/7 (7/7) | - | 0.1 | 0/7 (0/7) |
| LIG_AP2alpha_2 | DP[FW] | - | 0.021 | 4/7 (4/5) | I..F..D.F | 0.091 | 3/7 (3/5) |
| LIG_APCC_Dbox_1 | R..L..[IV] | - | 0.097 | 7/7 (4/4) | - | 0.1 | 0/7 (0/4) |
| LIG_APCC_KENbox_2 | KEN | KEN..P | 0.000000229 | 12/12 (10/10) | KEN | 0.0000637 | 12/12 (10/10) |
| LIG_BIR_III_3 | ^MA..F | - | 0.1 | 0/3 (0/3) | - | 0.1 | 0/3 (0/3) |
| LIG_BRCT_BRCA1_1 | SP.F | SP.F | 0.02 | 5/5 (5/5) | - | 0.1 | 0/5 (0/5) |
| LIG_Clathr_ClatBox_1 | L[IL].[FL][DE] | - | 0.026 | 7/14 (6/7) | - | 0.1 | 0/14 (0/7) |
| LIG_CRL4_Cdt2_1 | TDF....R | TDF | 0.013 | 3/3 (3/3) | TDF | 0.012 | 3/3 (3/3) |
| LIG_CtBP | P[ILM][DN]L[RS] | - | 0.00000232 | 15/18 (8/10) | - | 0.039 | 16/18 (9/10) |
| LIG_Dynein_DLC8_1 | K.TQT | S..K.TQT | 3.79E-08 | 7/7 (4/4) | S..K.TQT | 0.00000127 | 6/7 (4/4) |
| LIG_EH_1 | NPF | NPF | 3.47E-11 | 32/32 (12/12) | NPF | 6.31E-08 | 32/32 (12/12) |
| LIG_eIF4E_1 | Y....LL | - | 0.1 | 0/10 (0/3) | - | 0.1 | 0/10 (0/3) |
| LIG_EVH1_2 | PP..F | - | 0.1 | 0/7 (0/4) | - | 0.1 | 0/7 (0/4) |
| LIG_FHA_1 | T.E | - | 0.1 | 0/3 (0/3) | - | 0.1 | 0/3 (0/3) |
| LIG_HCF-1_HBM_1 | [DE]H.Y | DH.Y | 0.000000323 | 17/17 (7/7) | DH.Y | 0.00000144 | 17/17 (7/7) |
| LIG_HP1_1 | P.V.L | - | 0.1 | 0/7 (0/5) | - | 0.1 | 0/7 (0/5) |
| LIG_KEPE_1 | K.EP.E | IKQE..E | 0.000251 | 5/5 (4/4) | IKQE..E | 0.002 | 5/5 (4/4) |
| LIG_KEPE_2 | [ILV]K.EP | K.EP.E.D | 0.000000791 | 10/10 (10/10) | K.EP.E.D | 0.0000126 | 10/10 (10/10) |
| LIG_MAPK_2 | F.FP | F.FP | 0.005 | 5/5 (4/4) | F.FP | 0.039 | 5/5 (4/4) |
| LIG_NRBOX | L..LL | - | 0.1 | 0/9 (0/7) | - | 0.1 | 0/9 (0/7) |
| LIG_PCNA | Q.[ST][IL]..FF | Q.TL..FF | 7.63E-13 | 10/10 (6/6) | Q.TL..FF | 5.3E-12 | 10/10 (6/6) |
| LIG_PDZ_Class_1 | [EQ]T[DQRS][LV]$ | ET..$ | 3.73E-11 | 12/12 (10/10) | ET..$ | 1.54E-10 | 12/12 (10/10) |
| LIG_PP2B_1 | P.I.I | P.I.I | 0.002 | 6/6 (5/5) | P.I.I | 0.033 | 6/6 (5/5) |
| LIG_PTAP_UEV_1 | P[ST]AP[LPQS] | PTAP | 0.00000226 | 18/18 (13/13) | PTAP | 0.000221 | 14/18 (10/13) |
| LIG_PTB_Phospho_1 | NP.Y | NP.Y | 0.00016 | 9/10 (5/5) | NP.Y | 0.012 | 9/10 (5/5) |
| LIG_Rb_LxCxE_1 | L.C.[DE] | L.C.E | 0.00000161 | 24/30 (20/25) | L.C.E | 0.0000751 | 16/30 (12/25) |
| LIG_RGD | RGD | RGD | 0.000886 | 14/19 (8/9) | RGD | 0.038 | 14/19 (8/9) |
| LIG_SCF-TrCP1_1 | DSG | DSG..S | 8.79E-10 | 15/15 (12/12) | DSG..S | 5.55E-08 | 15/15 (12/12) |
| LIG_SH2_GRB2 | Y.N | A..Y.N | 0.00087 | 6/10 (6/6) | A..Y.N | 0.012 | 5/10 (5/6) |
| LIG_SH3_1 | P..P | - | 0.1 | 0/4 (0/3) | - | 0.1 | 0/4 (0/3) |
| LIG_SH3_3 | P..P | - | 0.1 | 0/6 (0/6) | - | 0.1 | 0/6 (0/6) |
| LIG_SIAH_1 | P.A.V.P | P.A.V.P | 7.06E-09 | 9/9 (8/8) | P.A.V.P | 0.000000232 | 9/9 (8/8) |
| LIG_TNKBM_1 | R..[AGP][DE]G | - | 0.00000523 | 9/13 (9/13) | - | 0.000662 | 9/13 (9/13) |
| LIG_TRAF6 | P.E..[DEY] | N.P.E..E..P | 0.005 | 4/8 (3/3) | N.P.E..E..P | 0.053 | 3/8 (3/3) |
| LIG_TRFH_1 | L.P | - | 0.1 | 0/3 (0/3) | - | 0.1 | 0/3 (0/3) |
| LIG_WW_1 | PPPY | PPPY | 0.051 | 4/11 (4/8) | - | 0.1 | 0/11 (0/8) |
| MOD_GSK3_1 | TPP.S | TPP.S | 0.081 | 3/3 (3/3) | - | 0.1 | 0/3 (0/3) |
| MOD_N-GLC_2 | N.C | - | 0.1 | 0/5 (0/4) | - | 0.1 | 0/5 (0/4) |
| MOD_NMyristoyl | ^MG...S | ^.G | 9.25E-08 | 11/11 (8/8) | ^.G | 0.00000108 | 11/11 (8/8) |
| MOD_PKA_1 | [KR]R.[ST] | RR.S | 0.0000124 | 14/19 (14/17) | RR.S | 0.000303 | 14/19 (14/17) |
| MOD_PKA_2 | R.S | RGS | 0.046 | 10/20 (9/18) | - | 0.1 | 0/20 (0/18) |
| MOD_PKB_1 | R.R..S | - | 0.1 | 0/4 (0/3) | - | 0.1 | 0/4 (0/3) |
| MOD_SUMO | [ILV]K.E | VK.E | 8.64E-08 | 28/28 (18/18) | VK.E | 0.000004 | 27/28 (18/18) |
| TRG_ER_diLys_1 | KK..$ | KK..$ | 0.000000866 | 6/8 (6/8) | KK..$ | 0.0000322 | 6/8 (6/8) |
| TRG_ER_KDEL_1 | DEL$ | KDEL$ | 0 | 11/11 (9/9) | KDEL$ | 0 | 11/11 (9/9) |
| TRG_LysEnd_APsAcLL_1 | [DE]...LL | - | 0.058 | 7/9 (6/7) | - | 0.1 | 0/9 (0/7) |
| TRG_NLS_Bipartite_1 | KR | - | 0.006 | 4/8 (4/7) | - | 0.087 | 4/8 (4/7) |
| TRG_NLS_MonoCore_2 | [KR][KR][PR][KR] | KR.R | 0.028 | 10/13 (9/11) | - | 0.1 | 0/13 (0/11) |
| TRG_NLS_MonoExtC_3 | [KR][KR]..[KLPR] | S.KRPR | 0.000723 | 5/15 (5/13) | - | 0.013 | 12/15 (11/13) |
| TRG_NLS_MonoExtN_4 | [KPR].[KR].[KR] | - | 0.045 | 6/12 (5/10) | - | 0.1 | 0/12 (0/10) |
| TRG_PTS1 | A.L$ | A.L$ | 0.001 | 3/3 (3/3) | A.L$ | 0.007 | 3/3 (3/3) |

1. The top-ranked pattern returned.
2. SLiMChance significance of top-ranked pattern.
3. Support for patterns in the top-ranked cloud, showing the number of sequences containing a motif as a proportion of the total number of sequences in the dataset. Figures in brackets are unrelated protein cluster (UPC) support, which corrects for evolutionary relationships.


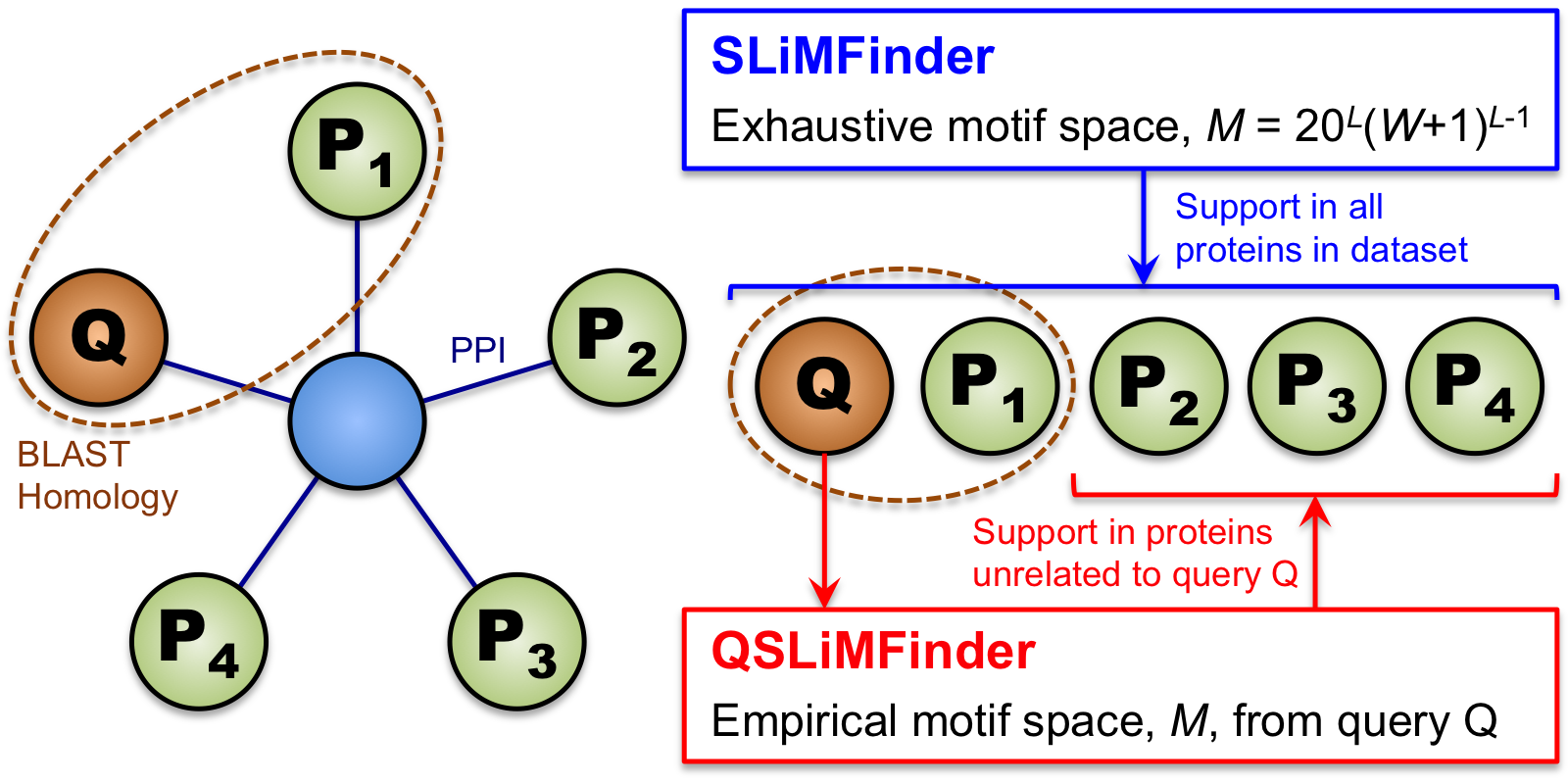


Supplementary Figure 1. The key difference between SLiMFinder and QSLiMFinder. Proteins P_1_ to P_4_ and query Q all interact with the central protein. In this example, P_1_ and Q share BLAST homology. SLiMFinder builds an exhaustive motif space of all possible patterns as constrained by SLiMBuild parameters, *L* (number of defined positions) and *W* (maximum wildcard spacer length between defined positions). Over-representation is based on the observed support of each pattern in the entire dataset. QSLiMFinder builds an empirical motif space from the query protein, Q, of all observed patterns constrained by SLiMBuild parameters. Q is then removed from the dataset and over-representation is based on observed support of each pattern in the remaining proteins. Proteins that can be linked to Q via (direct or indirect) BLAST homology relationships are also excluded.

###
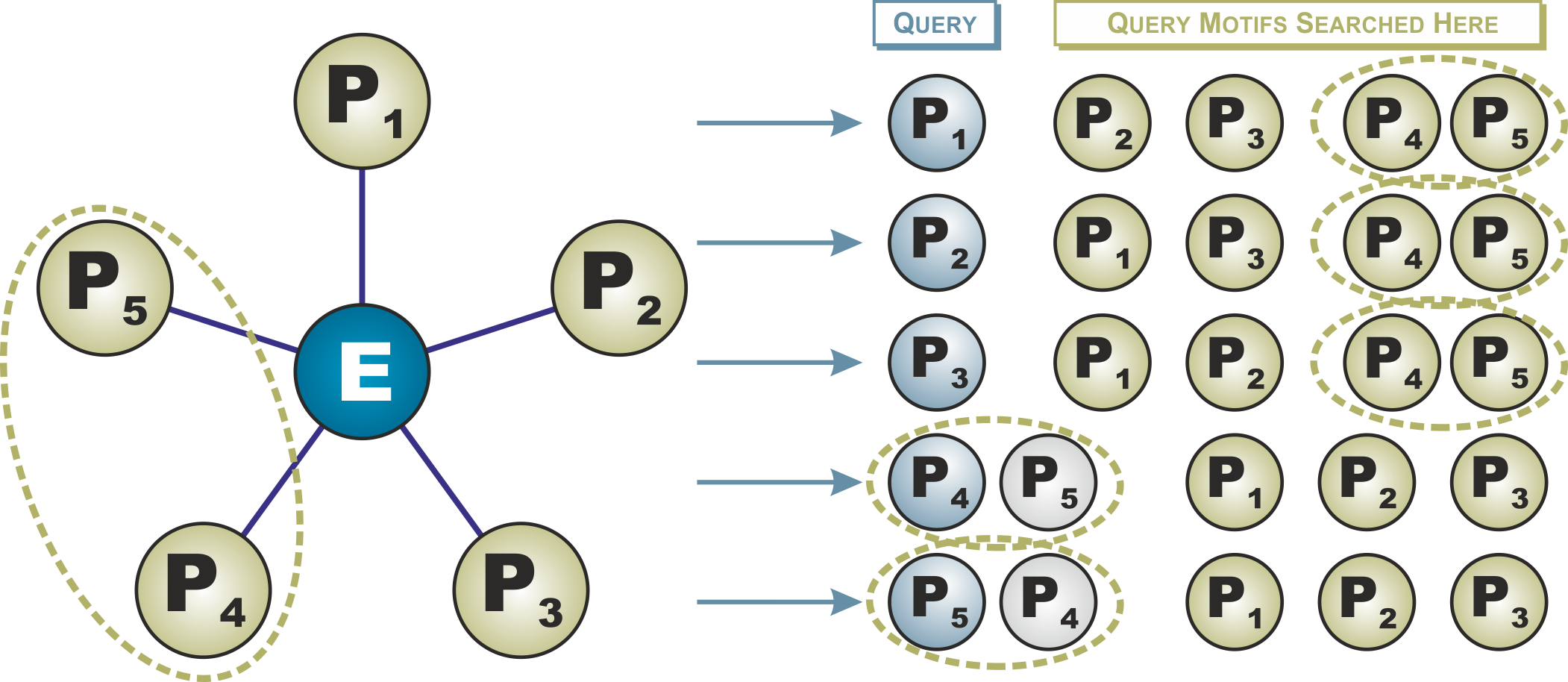


Supplementary Figure 2. Query and Search selection for ELM Benchmarking. Proteins P_1_ to P_5_ are all annotated in the ELM database as containing instances of the same motif that interacts with protein E. In this example, P_4_ and P_5_ share BLAST homology. Each protein is taken in turn to be the Query and any remaining unrelated proteins form the search dataset.
